# Design, synthesis, and apoptosis-promoting effect evaluation of novel pyrazole with benzo[*d*]thiazole derivatives containing aminoguanidine units

**DOI:** 10.1080/14756366.2019.1591391

**Published:** 2019-03-27

**Authors:** Da Chuan Liu, Mei Jia Gao, Qiang Huo, Tao Ma, Ying Wang, Cheng Zhu Wu

**Affiliations:** a School of Pharmacy, Bengbu Medical College, Bengbu, China;; b School of Public Foundation, Bengbu Medical College, Bengbu, China

**Keywords:** Synthesis, benzothiazole, pyrazoles, aminoguanidine, anticancer, apoptosis

## Abstract

New pyrazole with benzo[*d*]thiazoles containing hydrazinecarboximidamide substituent was synthesised and evaluated for cytotoxicity and apoptotic activity using the MTT assay, flow cytometry, and Western blot analysis. Among the compounds studied, (*E*)-2-((1-(6-((4-fluorobenzyl)oxy)benzo[*d*]thiazol-2-yl)-3-phenyl-1*H*- pyrazol-4-yl)methylene) hydrazinecarboximidamide (**8l**) was potent, with IC_50_ values of 2.41 µM, 2.23 µM, 3.75 µM and 2.31 µM *in vitro* anti-proliferative activity testing against triple-negative breast cancer cell line MDA-MB-231, non-triple-negative breast cancer MCF-7 cells, and human hepatocarcinoma HepG2 cells, and SMMC-7721 cells, respectively. Especially, the activity against MDA-MB-231 was similar to that of Doxorubicin, which was used as a positive control in this study. Next, the Annexin V/PI flow cytometry assay was used at different concentrations of compound **8l** to demonstrate that compound **81** induced apoptosis of MDA-MB-231 cells in a concentration-dependent manner. Finally, these results were further verified by Western blot analysis. Taken together, the results of this study revealed that compound **8l** may be a potential anticancer compound play a significant role in the subsequent researches.

## 1. Introduction

Despite the continued improvement in cancer therapy, cancer has remained a major public health problem worldwide, and an increasing number of patients are being diagnosed with cancer annually. According to the World Health Organisation (WHO), an estimated 9.0 million deaths occurred due to cancer in 2018, accounting for 22% of deaths of all non-communicable diseases (NCDs), thereby indicating that cancer accounts for nearly one in every four deaths in the world. Moreover, this percentage is likely to increase by 50% in the year 2020, which includes 15 million casualties, and ultimately reaches as many as 22.2 million cases by 2030^1^^,^^2^. The lungs, bronchus, breast, prostate, and colon continue to be the most common causes of cancer and life ends in death^3^. Populations in developing countries affected by numbers of different types of cancer in Africa, Asia, Central and South America account for more than 60% of the world's total cases of cancer, which results in about 70% of cancer deaths in the world^4^. The number of new cancer cases in China reached 4.29 million in 2015 and the 5-year survival of these patients was 36.9%^5^. Currently, there is no effective treatment for cancer patients in the clinic, however, chemotherapy is still the most widely used type of cancer treatment. Identifying novel, effective and safe chemotherapeutic agents for cancer treatment is one of the key challenges that are of utmost importance.

**Figure 1.**
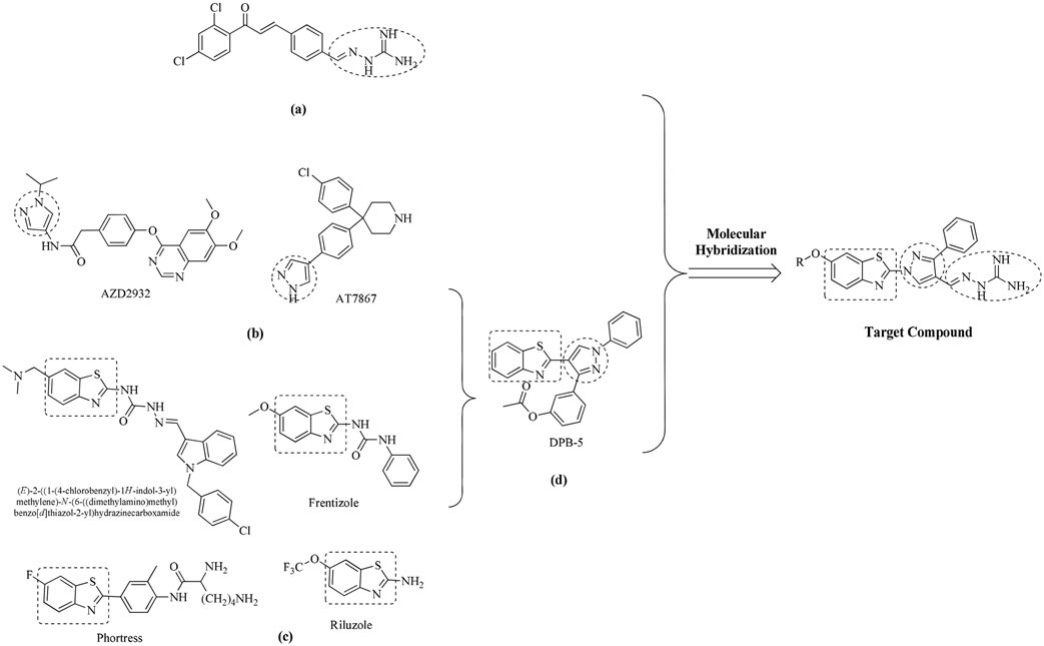
Rational design of the target compounds. (a) Structures of the previously reported compound with aminoguanidine. (b) Examples of pyrazole derivatives with biological activity. (c) Representative examples of benzothiazole derivatives. (d) A representative example of benzothiazole molecule with pyrazole that exhibits anticancer activity.

Aminoguanidine derivatives have recently been the focus of numerous studies because of their diverse range of biological properties, including their antibacterial^6^, antifungal^7^ anti-inflammatory^8^, and antimicrobial activities^9^. In addition, in previous studies, researchers have reported that the development of a series of chalcone with aminoguanidine derivatives showed some activity in the inhibition of tumour cells (such as HeLa and Hep3B)^10^, however, their activity was not high enough (8.7 µg/mL and 15.3 µg/mL respectively). Pyrazoles occupy a distinct niche in heterocyclic chemistry and represent a key motif in medicinal chemistry because of their capability to exhibit an array of bioactivities, including antimicrobial^11^^,^^12^_,_ anticancer^13^^,^^14^, anti-inflammatory^15^, antidepressant^16^, anticonvulsant^17^_,_ and selective enzyme inhibitory^18^ activities. Furthermore, the benzothiazole nucleus is a unique scaffold that is promising for further molecular exploration, and for the synthesis of novel compounds. Literature surveys revealed that benzothiazole analogues are associated with diverse pharmacological effects^19–21^ (Figure 1(c)). Especially, the anticancer activity of benzothiazole with pyrazole has received increased attention^22^ (Figure 1(d)). Therefore, to improve the antitumor activities of this type of compounds, in this study a series of new compounds were designed and synthesised by reserving the aminoguanidine moiety, and replacing the chalcone moiety by 1-benzo[*d*]thiazole-3-phenyl-pyrazole (named 2-(3-phenyl-pyrazol-1-yl)benzo[*d*]thiazole). In these compounds, we simultaneously changed the substituents on the benzo[*d*]thiazole and investigated the effects of these compounds on anti-proliferative activity. The structure of target compounds is shown in Figure 1.

## 2. Experimental

### 2.1. Chemistry

All reagents and solvents were purchased from commercial sources. The degree of reactions was monitored by thin layer chromatography (TLC) on Merck pre-coated silica GF254 plates and visualised using a combination of UV. Melting points were determined in open capillary tubes and were uncorrected. ^1^H NMR and ^13 ^C NMR spectra were collected at room temperature on a PX400 spectrometer with TMS and solvent signals allotted as internal standards. Chemical shifts were reported in ppm (d). Mass spectra were obtained on an Agilent 1260–6221 TOF mass spectrometer (Agilent Technologies, USA).

#### 2.1.1. Synthesis of 6-hydroxy-2-aminobenzothiazole (2)

A mixture of 6-methoxy-2, 3-dihydrobenzo[d]thiazol-2-amine (**1**) (10 g, 55.56 mmol) and 40 ml of hydrobromic acid (48% water solution) was refluxed at 126 °C for 20 h. The mixture was allowed to cool to room temperature and neutralised with NaOH solution to pH 7–8. Then, the precipitate was filtered and washed with water. The filtrate was stirred with 100 ml hot water for 0.5 h and the remaining precipitate was filtered to yield a brown solid, compound **2**.

#### 2.1.2. General procedure for the synthesis of 6-alkoxy-2-aminobenzothiazoles (3a–o)

A mixture of compound **2** (2 g, 12 mmol), potassium carbonate (2 g, 14.4 mmol), appropriate alkyl bromide or benzyl chloride derivatives (1.32 mmol), and a catalytic amount of benzyltriethylamine chloride (TEBA) was heated in 50 ml acetonitrile under reflux for 24-48 h. After removing the solvent under reduced pressure, 80 ml of hot water was poured into the flask, and the mixture was stirred for 0.5 h to eliminate potassium carbonate excess. The remaining precipitate was filtered to yield a russet solid (**3a–o**), which was used without further purification.

#### 2.1.3. General procedure for the synthesis of 6-alkoxy-2-hydrazinobenzothiazoles (4a–o)

A mixture of compounds **3a–o** (20 mmol) and 0.6 ml of 98% H_2_SO_4_ solution (water solutions) in 20 ml of glycol was refluxed for 0.5 h at 80 °C. Then, 10 ml of hydrazine hydrate was added, and the mixture was heated at 140 °C for 5 h. After cooling to the room temperature, the mixture was added to 50 ml of ice-cold water. The precipitate formed was filtered and washed with water to obtain a light green needlelike solid compound **4a–o**.

#### 2.1.4. General procedure for the synthesis of 6-alkoxy-2-(2-(1-phenylethylidene) hydrazinyl)benzo[d]thiazole (6a–o)

Hydrazone derivatives were prepared by reacting compounds **4a–o** (5 mmol) with acetophenone (compound **5**, 5 mmol) in the presence of glacial acetic acid in ethanol. After that, the resulting reaction mixture was refluxed for 1 h. On cooling, a solid separated out which was filtered, dried and crystallized from ethanol to afford hydrazones, compound **6a–o** (about 80% yields).

#### 2.1.5. General procedure for the synthesis of 1–(6-alkoxybenzo[d]thiazol-2-yl)-3-phenyl-1H-pyrazole-4-carbaldehyde (7a–o)

To a cold, stirred solution of dimethylformamide (15 ml) and phosphorous oxychloride (40 mmol) was added hydrazone (compound **6**, 10 mmol). The reaction mixture was stirred at 55–60 °C for 5 h, then cooled to room temperature, poured into ice cold water and neutralised with saturated aqueous sodium bicarbonate solution whereupon a solid separated out that was filtered, washed with excess of cold water, dried and crystallized from acetic acid to afford aldehydes **7a–o**.

#### 2.1.6. General procedure for the synthesis of 2-((1–(6-alkoxybenzo[d]thiazol-2-yl) -3-phenyl-1H-pyrazol-4-yl)methylene)hydrazinecarboximidamide (8a–8o)

The compounds **7** (5 mmol) was reacted with aminoguanidine bicarbonate (5 mmol) in 20 ml refluxing ethanol in the presence of 5 drops of concentrated hydrochloric acid at 60–70 °C for 8–12 h. The solution was evaporated to dryness under reduced pressure, and the residue was purified by silica gel column chromatography with dichloromethane: methanol (50:1) to afforded white solid **8a–8o**.

The yield, melting point, analytical data and spectral data of each compound are given below.

##### (E)-2-((1-(6-methoxybenzo[d]thiazol-2-yl)-3-phenyl-1H-pyrazol-4-yl)methylene)hydrazinecarboximidamide (8a)

White powder, yield: 68%. mp: 283.5-285.5 °C. ^1^H NMR (600 MHz, dmso) *δ* 11.97 (s, 1H), 9.40 (s, 1H), 8.22 (s, 1H), 7.92 (s, 2H), 7.79 (d, *J* = 8.9 Hz, 1H), 7.69 (dd, *J* = 13.7, 4.6 Hz, 3H), 7.59 – 7.47 (m, 3H), 7.12 (dd, *J* = 8.9, 2.5 Hz, 1H), 3.82 (s, 3H, -OCH_3_). ^13 ^C NMR (151 MHz, dmso) *δ* 157.78, 157.56, 155.66, 153.83, 144.85, 138.68, 134.79, 131.13, 129.81, 129.43 (2C), 128.77 (2C), 128.37, 123.13, 118.76, 116.43, 106.03, 56.23. HR-ESI-MS: *m/z* [M + H]^+^ calcd for C_19_H_18_N_7_OS: 392.1288; found: 392.1293.

##### (E)-2-((3-phenyl-1-(6-propoxybenzo[d]thiazol-2-yl)-1H-pyrazol-4-yl)methylene)hydrazinecarboximidamide (8b)

White powder, yield: 64%. mp: 280.3-282.3 °C. ^1^H NMR (600 MHz, dmso) *δ* 12.02 (s, 1H), 9.40 (s, 1H), 8.22 (s, 1H), 7.95 (s, 2H), 7.77 (d, *J* = 8.9 Hz, 1H), 7.71 – 7.62 (m, 3H), 7.58 – 7.46 (m, 3H), 7.10 (dd, *J* = 8.9, 2.4 Hz, 1H), 3.97 (t, *J* = 6.5 Hz, 2H), 1.86 – 1.63 (m, 2H), 0.97 (t, *J* = 7.4 Hz, 3H). ^13 ^C NMR (151 MHz, dmso) *δ* 157.51, 157.15, 155.67, 153.78, 144.74, 138.62, 134.76, 131.13, 129.80, 129.44 (2C), 128.76 (2C), 128.34, 123.11, 118.75, 116.76, 106.61, 70.12, 22.44, 10.81. HR-ESI-MS: *m/z* [M + H]^+^ calcd for C_21_H_22_N_7_OS: 420.1601; found: 420.1606.

##### (E)-2-((1-(6-butoxybenzo[d]thiazol-2-yl)-3-phenyl-1H-pyrazol-4-yl)methylene)hydrazinecarboximidamide (8c)

White powder, yield: 60%. mp: 271.3-272.9 °C. ^1^H NMR (600 MHz, dmso) *δ* 11.93 (s, 1H), 9.40 (s, 1H), 8.22 (s, 1H), 7.91 (s, 2H), 7.78 (d, *J* = 8.9 Hz, 1H), 7.70 – 7.65 (m, 3H), 7.57 – 7.48 (m, 3H), 7.11 (dd, *J* = 8.9, 2.5 Hz, 1H), 4.02 (t, *J* = 6.5 Hz, 2H), 1.77 – 1.65 (m, 2H), 1.43 (dd, *J* = 14.9, 7.5 Hz, 2H), 0.92 (t, *J* = 7.4 Hz, 3H). ^13 ^C NMR (151 MHz, dmso) *δ* 157.51, 157.19, 155.63, 153.81, 144.75, 138.72, 134.77, 131.13, 129.81, 129.44 (2C), 128.76 (2C), 128.36, 123.11, 118.74, 116.79, 106.63, 68.37, 31.13, 19.17, 14.11. HR-ESI-MS: *m/z* [M + H]^+^ calcd for C_22_H_24_N_7_OS: 434.1758; found: 434.1761.

##### (E)-2-((1-(6-(pentyloxy)benzo[d]thiazol-2-yl)-3-phenyl-1H-pyrazol-4-yl)methylene)hydrazinecarboximidamide (8d)

White powder, yield: 65%. mp: 264.6-266.2 °C. ^1^H NMR (600 MHz, dmso) *δ* 11.98 (s, 1H), 9.40 (s, 1H), 8.22 (s, 1H), 7.95 (s, 2H), 7.77 (d, *J* = 8.9 Hz, 1H), 7.70 – 7.63 (m, 3H), 7.58 – 7.48 (m, 3H), 7.10 (dd, *J* = 8.9, 2.5 Hz, 1H), 4.00 (t, *J* = 6.5 Hz, 2H), 1.77 – 1.68 (m, 2H), 1.44 – 1.28 (m, 4H), 0.88 (t, *J* = 7.2 Hz, 3H). ^13 ^C NMR (151 MHz, dmso) *δ* 157.50, 157.17, 155.65, 153.79, 144.74, 138.64, 134.76, 131.13, 129.80, 129.44 (2C), 128.76 (2C), 128.34, 123.10, 118.75, 116.76, 106.58, 68.63, 28.77, 28.14, 22.33, 14.36. HR-ESI-MS: *m/z* [M + H]^+^ calcd for C_23_H_26_N_7_OS: 448.1914; found: 448.1915.

##### (E)-2-((1-(6-(hexyloxy)benzo[d]thiazol-2-yl)-3-phenyl-1H-pyrazol-4-yl)methylene)hydrazinecarboximidamide (8e)

White powder, yield: 76%. mp: 254.4-255.8 °C. ^1^H NMR (600 MHz, dmso) *δ* 11.97 (s, 1H), 9.40 (s, 1H), 8.22 (s, 1H), 7.99 (s, 2H), 7.77 (d, *J* = 8.9 Hz, 1H), 7.71 – 7.63 (m, 3H), 7.58 – 7.49 (m, 3H), 7.10 (dd, *J* = 8.9, 2.5 Hz, 1H), 4.00 (t, *J* = 6.5 Hz, 2H), 1.81 – 1.63 (m, 2H), 1.44 – 1.36 (m, 2H), 1.34 – 1.21 (m, 4H), 0.85 (dd, *J* = 9.2, 4.8 Hz, 3H). ^13 ^C NMR (151 MHz, dmso) *δ* 157.50, 157.17, 155.64, 153.80, 144.74, 138.66, 134.76, 131.13, 129.81, 129.44 (2C), 128.76 (2C), 128.35, 123.10, 118.75, 116.76, 106.59, 68.65, 31.43, 29.04, 25.62, 22.52, 14.35. HR-ESI-MS: *m/z* [M + H]^+^ calcd for C_23_H_26_N_7_OS: 462.2070; found: 462.2066.

##### (E)-2-((1-(6-(heptyloxy)benzo[d]thiazol-2-yl)-3-phenyl-1H-pyrazol-4-yl)methylene)hydrazinecarboximidamide (8f)

White powder, yield: 70%. mp: 248.8-250.2 °C. ^1^H NMR (600 MHz, dmso) *δ* 11.95 (s, 1H), 9.41 (s, 1H), 8.22 (s, 1H), 7.91 (s, 2H), 7.77 (d, *J* = 8.9 Hz, 1H), 7.68 (dd, *J* = 4.6, 1.8 Hz, 3H), 7.53 (ddd, *J* = 8.5, 7.7, 2.2 Hz, 3H), 7.10 (dd, *J* = 8.9, 2.5 Hz, 1H), 4.00 (t, *J* = 6.5 Hz, 2H), 1.82 – 1.65 (m, 2H), 1.39 (dd, *J* = 15.2, 7.6 Hz, 2H), 1.34 – 1.18 (m, 6H), 0.84 (t, *J* = 6.9 Hz, 3H). ^13 ^C NMR (151 MHz, dmso) *δ* 157.51, 157.18, 155.63, 153.80, 144.74, 138.68, 134.77, 131.13, 129.81, 129.44 (2C), 128.76 (2C), 128.35, 123.10, 118.75, 116.77, 106.60, 68.64, 31.69, 29.08, 28.87, 25.92, 22.49, 14.38. HR-ESI-MS: *m/z* [M + H]^+^ calcd for C_25_H_30_N_7_OS: 476.2227; found: 476.2211.

##### (E)-2-((1-(6-(octyloxy)benzo[d]thiazol-2-yl)-3-phenyl-1H-pyrazol-4-yl)methylene)hydrazinecarboximidamide (8g)

White powder, yield: 50%. mp: 245.0-246.4 °C. ^1^H NMR (600 MHz, dmso) *δ* 12.02 (s, 1H), 9.40 (s, 1H), 8.22 (s, 1H), 7.92 (s, 2H), 7.77 (d, *J* = 8.9 Hz, 1H), 7.70 – 7.64 (m, 3H), 7.59 – 7.48 (m, 3H), 7.10 (dd, *J* = 8.9, 2.6 Hz, 1H), 4.00 (t, *J* = 6.5 Hz, 2H), 1.75 – 1.67 (m, 2H), 1.38 (dd, *J* = 15.2, 7.5 Hz, 2H), 1.33 – 1.16 (m, 8H), 0.83 (dd, *J* = 8.7, 5.2 Hz, 3H). ^13 ^C NMR (151 MHz, dmso) *δ* 157.50, 157.17, 155.68, 153.79, 144.74, 138.62, 134.76, 131.13, 129.80, 129.44 (2C), 128.76 (2C), 128.35, 123.10, 118.76, 116.77, 106.59, 68.64, 31.68, 29.27 – 28.97 (m), 25.95, 22.52, 14.38. HR-ESI-MS: *m/z* [M + H]^+^ calcd for C_26_H_32_N_7_OS: 490.2384; found: 490.2377.

##### (E)-2-((1-(6-(nonyloxy)benzo[d]thiazol-2-yl)-3-phenyl-1H-pyrazol-4-yl)methylene)hydrazinecarboximidamide (8h)

White powder, yield: 58%. mp: 242.7-244.2 °C. ^1^H NMR (600 MHz, dmso) *δ* 11.96 (s, 1H), 9.40 (s, 1H), 8.22 (s, 1H), 7.90 (s, 2H), 7.77 (d, *J* = 8.9 Hz, 1H), 7.67 (dd, *J* = 7.9, 1.5 Hz, 3H), 7.57 – 7.45 (m, 3H), 7.09 (dd, *J* = 8.9, 2.5 Hz, 1H), 3.99 (t, *J* = 6.5 Hz, 2H), 1.76 – 1.64 (m, 2H), 1.38 (dd, *J* = 15.1, 7.6 Hz, 2H), 1.33 – 1.27 (m, 2H), 1.27 – 1.08 (m, 8H), 0.82 (t, *J* = 6.9 Hz, 3H). ^13 ^C NMR (151 MHz, dmso) *δ* 157.50, 157.18, 155.66, 153.80, 144.75, 138.67, 134.77, 131.14, 129.80, 129.43 (2C), 128.75 (2C), 128.35, 123.10, 118.75, 116.77, 106.60, 68.65, 31.71, 29.41, 29.28 – 29.00 (m), 25.94, 22.52, 14.37. HR-ESI-MS: *m/z* [M + H]^+^ calcd for C_23_H_26_N_7_OS: 504.2540; found: 504.2536.

##### (E)-2-((1-(6-(benzyloxy)benzo[d]thiazol-2-yl)-3-phenyl-1H-pyrazol-4-yl)methylene)hydrazinecarboximidamide (8i)

White powder, yield: 76%. mp: 248.5-249.8 °C. ^1^H NMR (600 MHz, dmso) *δ* 11.99 (s, 1H), 9.41 (s, 1H), 8.22 (s, 1H), 7.93 (s, 2H), 7.81 (d, *J* = 9.0 Hz, 2H), 7.70 – 7.65 (m, 2H), 7.53 (dt, *J* = 7.1, 4.9 Hz, 3H), 7.46 (d, *J* = 7.6 Hz, 2H), 7.39 (t, *J* = 7.5 Hz, 2H), 7.33 (t, *J* = 7.3 Hz, 1H), 7.20 (dd, *J* = 8.9, 2.5 Hz, 1H), 5.16 (s, 2H). ^13 ^C NMR (151 MHz, dmso) *δ* 157.71, 156.82, 155.67, 153.85, 145.03, 138.67, 137.13, 134.73, 131.12, 129.82, 129.44 (2C), 128.91 (2C), 128.77 (2C), 128.41, 128.40, 128.25 (2C), 123.18, 118.80, 116.94, 107.27, 70.40. HR-ESI-MS: *m/z* [M + H]^+^ calcd for C_25_H_22_N_7_OS: 468.1601; found: 468.1603.

##### (E)-2-((1-(6-((2-fluorobenzyl)oxy)benzo[d]thiazol-2-yl)-3-phenyl-1H-pyrazol-4-yl)methylene)hydrazinecarboximidamide (8j)

White powder, yield: 55%. mp: 263.6-264.2 °C. ^1^H NMR (600 MHz, dmso) *δ* 12.07 (s, 1H), 9.42 (s, 1H), 8.23 (s, 1H), 7.95 (s, 2H), 7.85 (d, *J* = 2.6 Hz, 1H), 7.82 (d, *J* = 8.9 Hz, 1H), 7.68 (dd, *J* = 8.0, 1.4 Hz, 2H), 7.59 (dd, *J* = 12.0, 4.5 Hz, 1H), 7.56 – 7.49 (m, 3H), 7.45 – 7.38 (m, 1H), 7.28 – 7.20 (m, 3H), 5.21 (s, 2H). ^13 ^C NMR (151 MHz, dmso) *δ* 161.68, 160.05, 157.86, 156.64, 155.73, 153.87, 145.20, 138.60, 134.75, 131.20 – 131.10 (m), 130.96 (d), 129.82, 129.44 (2C), 128.77 (2C), 128.42, 125.00 (d), 123.96 (d), 123.22, 118.83, 116.90, 115.93, 115.80, 107.31, 64.78. HR-ESI-MS: *m/z* [M + H]^+^ calcd for C_25_H_21_FN_7_OS: 486.1507; found: 486.1503.

##### (E)-2-((1-(6-((3-fluorobenzyl)oxy)benzo[d]thiazol-2-yl)-3-phenyl-1H-pyrazol-4-yl)methylene)hydrazinecarboximidamide (8k)

White powder, yield: 79%. mp: 259.4-260.5 °C. ^1^H NMR (600 MHz, dmso) *δ* 11.95 (s, 1H), 9.41 (s, 1H), 8.22 (s, 1H), 7.94 (s, 2H), 7.81 (dd, *J* = 10.0, 5.7 Hz, 2H), 7.68 (dd, *J* = 8.0, 1.3 Hz, 2H), 7.57 – 7.50 (m, 3H), 7.47 – 7.41 (m, 1H), 7.35 – 7.26 (m, 2H), 7.22 (dd, *J* = 8.9, 2.6 Hz, 1H), 7.18 – 7.12 (m, 1H), 5.20 (s, 2H). ^13 ^C NMR (151 MHz, dmso) *δ* 163.45, 161.84, 157.82, 156.58, 155.64, 153.88, 145.15, 140.09 (d), 138.69, 134.73, 131.18-130.83 (t), 129.83, 129.44 (2C), 128.76 (2C), 128.41, 124.05 (d), 123.22, 118.81, 116.93, 115.15 (d), 114.77 (d), 107.37, 69.52. HR-ESI-MS: *m/z* [M + H]^+^ calcd for C_25_H_21_FN_7_OS: 486.1507; found: 486.1502.

##### (E)-2-((1-(6-((4-fluorobenzyl)oxy)benzo[d]thiazol-2-yl)-3-phenyl-1H-pyrazol-4-yl)methylene)hydrazinecarboximidamide (8l)

White powder, yield: 64%. mp: 253.5-254.7 °C. ^1^H NMR (600 MHz, dmso) *δ* 11.94 (s, 1H), 9.41 (s, 1H), 8.22 (s, 1H), 7.94 (s, 2H), 7.81 (t, *J* = 5.7 Hz, 2H), 7.68 (dd, *J* = 8.0, 1.3 Hz, 2H), 7.61 – 7.45 (m, 5H), 7.29 – 7.12 (m, 3H), 5.15 (s, 2H). ^13 ^C NMR (151 MHz, dmso) *δ* 163.09, 161.48, 157.75, 156.71, 155.64, 153.87, 145.07, 138.70, 134.73, 133.37 (d), 131.12, 130.53 (d), 129.83, 129.44 (2C), 128.77 (2C), 128.41, 123.19, 118.81, 116.96, 115.81, 115.66, 107.30, 69.68. HR-ESI-MS: *m/z* [M + H]^+^ calcd for C_25_H_21_FN_7_OS: 486.1507; found: 486.1507.

##### (E)-2-((1-(6-((2-chlorobenzyl)oxy)benzo[d]thiazol-2-yl)-3-phenyl-1H-pyrazol-4-yl)methylene)hydrazinecarboximidamide (8m)

White powder, yield: 79%. mp: 246.4-247.5 °C. ^1^H NMR (600 MHz, dmso) *δ* 11.97 (s, 1H), 9.45 (s, 1H), 8.24 (s, 1H), 7.93 (s, 2H), 7.82 (d, *J* = 5.7 Hz, 2H), 7.69 (dd, *J* = 7.8, 1.4 Hz, 2H), 7.67 – 7.52 (m, 5H), 7.47 – 7.40 (m, 1H), 7.39 – 7.12 (m, 3H), 5.19 (s, 2H). ^13 ^C NMR (600 MHz, dmso) *δ* 157.81, 156.34, 155.54, 153.87, 145.26, 139.73, 138.67, 134.73, 133.65, 131.11, 129.82, 129.81, 129.44 (2C), 129.42, 128.77 (2C), 128.71, 128.64, 128.30, 123.27, 118.79, 116.95, 107.21, 68.97. HR-ESI-MS: *m/z* [M + H]^+^ calcd for C_25_H_21_ClN_7_OS: 502.1211; found: 502.1215.

##### (E)-2-((1-(6-((3-chlorobenzyl)oxy)benzo[d]thiazol-2-yl)-3-phenyl-1H-pyrazol-4-yl)methylene)hydrazinecarboximidamide (8n)

White powder, yield: 78%. mp: 248.0.5-248.8 °C. ^1^H NMR (600 MHz, dmso) *δ* 11.95 (s, 1H), 9.41 (s, 1H), 8.22 (s, 1H), 7.93 (s, 2H), 7.80 (dd, *J* = 11.2, 5.7 Hz, 2H), 7.67 (d, *J* = 6.7 Hz, 2H), 7.57 – 7.50 (m, 3H), 7.47 – 7.32 (m, 3H), 7.21 (dd, *J* = 8.9, 2.5 Hz, 1H), 5.18 (s, 2H). ^13 ^C NMR (151 MHz, dmso) *δ* 157.82, 156.54, 155.64, 153.87, 145.16, 139.73, 138.67, 134.73, 133.59, 131.10, 130.85, 129.82, 129.44 (2C), 128.76 (2C), 128.40, 128.32, 127.83, 126.70, 123.22, 118.81, 116.90, 107.36, 69.44. HR-ESI-MS: *m/z* [M + H]^+^ calcd for C_25_H_21_ClN_7_OS: 502.1211; found: 502.1216.

##### (E)-2-((1-(6-((2,4-dichlorobenzyl)oxy)benzo[d]thiazol-2-yl)-3-phenyl-1H-pyrazol-4-yl)methylene)hydrazinecarboximidamide (8o)

White powder, yield: 67%. mp: 262.4-263.6 °C. ^1^H NMR (600 MHz, dmso) *δ* 11.86 (s, 1H), 9.43 (s, 1H), 8.22 (s, 1H), 7.92 (s, 2H), 7.84 (d, *J* = 11.3 Hz, 2H), 7.68 (d, *J* = 6.9 Hz, 3H), 7.64 (d, *J* = 8.2 Hz, 1H), 7.60 – 7.50 (m, 3H), 7.48 (d, *J* = 7.2 Hz, 1H), 7.23 (dd, *J* = 8.8, 1.9 Hz, 1H), 5.21 (s, 2H). ^13 ^C NMR (151 MHz, dmso) *δ* 157.99, 156.45, 155.57, 153.92, 145.33, 138.76, 134.78, 134.09, 134.08, 133.68, 131.81, 131.10, 129.85, 129.46 (2C), 129.40, 128.78 (2C), 128.44, 128.03, 123.27, 118.83, 116.93, 107.41, 67.49. HR-ESI-MS: *m/z* [M + H]^+^ calcd for C_25_H_20_FN_7_OS: 536.0822; found: 536.0823.

### 2.2. Biological evaluation

#### 2.2.1. Cell culture, growth conditions, and treatments

SMMC7721 (human liver carcinoma cells), HepG2 (human liver hepatocellular carcinoma cells) and SW480 (human colorectal carcinoma cells) were cultured in Dulbecco’s modified Eagle’s medium (DMEM), which was obtained from Gibco (Grand Island, NY, USA). MDA-MB-231 cells (human breast cancer cells) were cultured in minimum essential medium (MEM, also obtained from Gibco) supplemented with 10% foetal bovine serum (FBS, Millipore, USA) and maintained at 37 °C in a humidified incubator with 5% CO_2_. All four types of cancer cells were obtained from Shanghai Cell Bank, Chinese Academy of Sciences (CAS). To establish a hypoxic condition, the culture medium was supplemented with 150 µmol/l CoCl_2_ for 24 h. The compounds were dissolved in dimethyl sulfoxide (DMSO, BIOSHARP, Hefei, China). The PX-478 (Selleck Chemicals, Houston, USA), a selective HIF-1α inhibitor was dissolved in ddH_2_O. The control group was treated with DMSO only under identical conditions.

#### 2.2.2. In vitro anti-proliferative activity

The 4 kinds of tested cells were seeded in 96-well microtiter plates at a density of 5 × 10^3^ cells per well and treated with different concentrations of the compounds and adriamycin for 48 h. At the end of the incubation period, 15 µL of MTT (purchased from Sigma USA), at a dose of 5 mg/mL in phosphate-buffered saline (PBS), was added to each well and incubated at 37 °C for 4 h. After 4 h, the MTT solution was replaced with 150 µL of DMSO to dissolve the formazan crystals. The plates were further incubated for 30 min, and the cell viability was determined by measuring absorbance at a test wavelength of 490 nm with the microplate reader (Synergy HT, BioTek, Vermont, USA).

#### 2.2.3. Analysis of the apoptosis by flow cytometry

MDA-MB-231 cells were plated in 6-well plates (5.0 × 10^5^ cells per well) and incubated at 37 °C for 24 h. Exponentially growing cells were then incubated with compound **8l** at different concentrations (0, 1, 2, 4 and 8 µM). Following 24 h of incubation, untreated cells (control) and cells treated with compound **8l** were centrifuged at 1200 rpm for 10 min, then collected and washed twice with PBS, once with 500 µL Binding Buffer, and stained with 5 µL annexin V-FITC and 5 µL PI at room temperature in the dark. Apoptotic cells were quantified using a FACSCalibur flow cytometer with the Cell Quest software (Becton-Dickinson, Franklin Lakes, NJ).

#### 2.2.4. Western blotting assay

MDA-MB-231 cells were collected after being treated with **8l** at different concentrations (1.2 µM, half of the IC_50_; 2.4 µM, the IC_50_; 4.8 µM, double of the IC_50_) for 48 h. Total cell lysates were prepared in RIPA buffer supplemented with protease inhibitors. The proteins were fractionated with 10% sodium dodecyl sulphate polyacrylamide gel electrophoresis (SDS PAGE) and electroblotted onto nitrocellulose membrane (Bio-Rad). The membranes were probed with primary antibodies and then probed with relative secondary antibody. β-Actin was used as a loading control.

## 3. Results and discussion

### 3.1. Chemistry

**Scheme 1.**
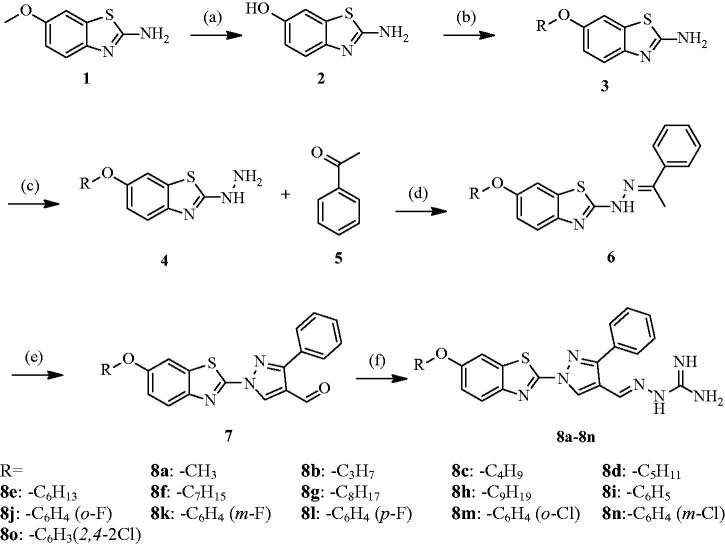
Synthesis of compounds **8a–8o**. Reagent and conditions: (a) HBr (48% in H_2_O), reflux, 18 h; (b) RBr/RPhCH_2_Cl, CH_3_COCH_3_, K_2_CO_3_, reflux, 20 h; (c) 98%H_2_SO_4_, (CH_2_OH)_2_, 80 °C, 0.5 h, NH_2_NH_2_H_2_O, 140 °C, 5 h; (d) EtOH, AcONa, reflux, 1 h; (e) POCl_3_, DMF, 55–60 °C, 5 h; (f) aminoguanidine bicarbonate, EtOH, 60–70 °C, 8–12 h.

The synthetic procedure adopted to obtain the target compounds is shown in Scheme 1. The starting material, 6-methoxy-1, 3-benzothiazol-2-amine (compound **1**), reacted with hydrobromic acid (48% water solutions) to obtain compound **2**^23^. Compound **2** reacted further with appropriate alkyl bromides and substituted phenol in acetone to obtain derivative **3**^24^. Subsequently, compound **3** was treated with hydrazine hydrate in the presence of sulphuric acid (98% water solutions) to create hydrazines derivatives **4**^25^. Then hydrazone derivatives (compound **6**) were prepared by reaction of acetophenone **5** with hydrazines derivatives **4** in the presence of glacial acetic acid in ethanol. Next, compound **6** reacted under Vilsmeier–Haack (DMF–POCl_3_) conditions and afforded corresponding pyrazole-4-carbaldehyde derivatives **7**^26^. Compounds **7** then reacted with aminoguanidine bicarbonate in the presence of catalytic amounts of hydrochloric acid in ethanol to provide the compounds in series **8a–o**^13^. The chemical structures were characterised using ^1^H NMR, ^13 ^C NMR, and HR-ESI-MS. A detailed overview of physical and analytical data is provided in the experimental procedures section.

### 3.2. Biological evaluation

#### 3.2.1. In vitro cytotoxicity against human cancer cell lines

**Figure 2.**
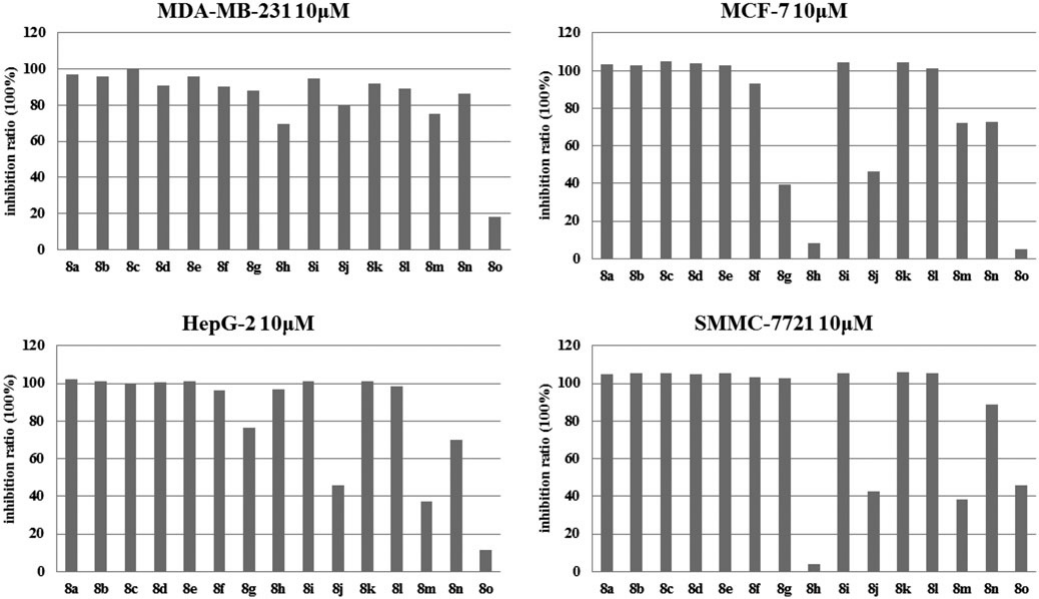
The comparison of the inhibitory activity of synthesised compounds against four cell lines at 10 μM in the preliminary screening test. The ordinate represents inhibition ratio (%), the abscissa is the synthesised compounds.

**Figure 3.**
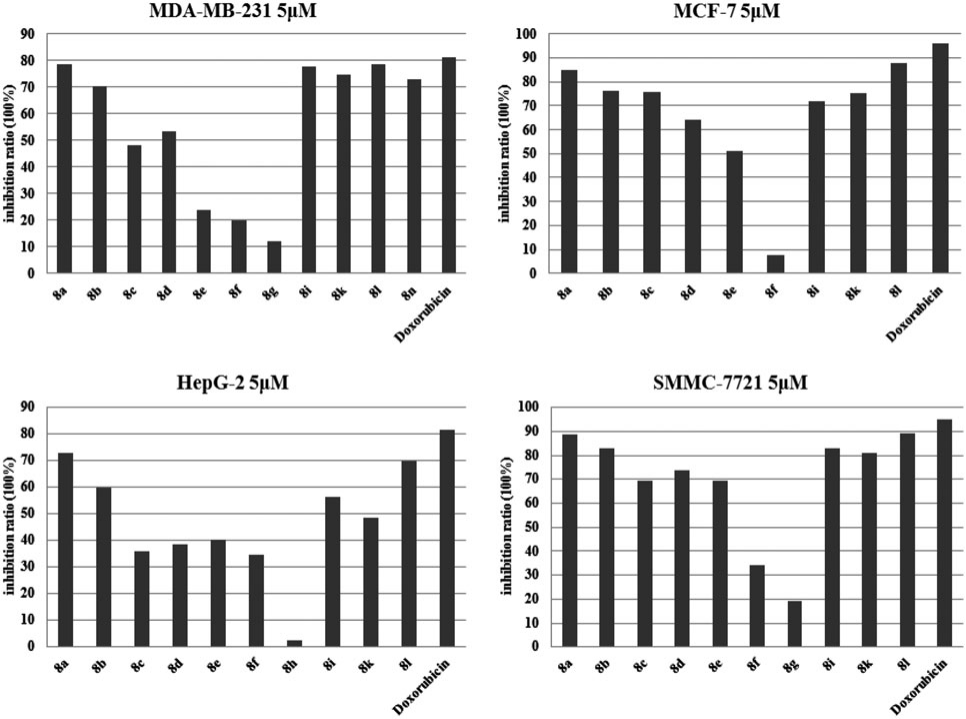
The inhibitory activity of the compounds with higher inhibition ratio against four cell lines at 5 μM in the screening test. The ordinate represents inhibition ratio (%) and the abscissa represents the chosen compounds and the reference drug Doxorubicin.

All synthesised compounds **8a–o** were evaluated for their anticancer activities against four human cancer cell lines, which included triple-negative breast cancer cell line MDA-MB-231, non-triple-negative breast cancer cells MCF**-**7, and two types of human hepatocarcinoma cell lines, HepG2 and SMMC-7721. The clinically used antineoplastic drug Doxorubicin was used as the reference drug. Figure 2 shows that in the preliminary screening test, synthesised compounds showed moderate to significant inhibitory activity against all four cell lines at a dose of 10 µM. Among them, compounds **8a**–**8i**, **8k**, **8 l,** and **8n** had better inhibitory activities against one or more type of cancer cells. Next, compounds with a higher inhibition ratio were chosen to be screened at lower concentrations based on the data presented in Figure 3. By analogy, we decided to pick some compounds with better anticancer activities. With respect to the four human cancer cell lines, the IC_50_ for the chosen compounds was calculated, and the results are summarised in Table 1. The values presented in Table 1 represent the concentration at which a 50% decrease in cell growth was observed after 48 h of incubation in the presence of the drug, and compared with control cells that were treated with DMSO. Moreover, among the compounds tested, compounds **8a** and **8l** displayed better activity then others against all four human cancer cell lines. However, most other compounds, which shared very similar structures, did not show any significant anticancer activity at low concentrations, thereby suggesting that their anticancer activities were sensitive to the structural perturbations. Among them, compound **8a** showed better activity against human hepatocarcinoma cell lines, HepG2 and SMMC-7721, with IC_50_ values of 3.34 µM and 2.03 µM, respectively. In addition, compound **8l** also exhibited anticancer activity with IC_50_ values against HepG2 and SMMC-7721of 3.75 µM and 2.31 µM, respectively. Notably, compound **8l** had a higher activity against MDA-MB-231 and MCF**-**7 cells with IC_50_ values of 2.41 µM and 2.23 µM, respectively. Especially, when triple-negative breast cancer cell MDA-MB-231 was evaluated, compound **8l** displayed a remarkable activity, which was as good as the clinically used antineoplastic drug Doxorubicin. However, compound **8l** also had toxicity towards normal cells (MCF-10A at 10 µM), thus, how to reduce the toxicity of compound **8l** was an unsolved problem as well as the future study direction.

**Table 1.** *In vitro* anticancer activity of the chosen compounds against four cancer cell lines^a^ (IC_50_ μM^b^).

Compound	MDA-MB-231	MCF-7	HepG-2	SMMC-7721
**8a**	3.07 ± 0.30	3.25 ± 0.45	**3.45 ± 0.28**^c^	**2.03 ± 0.36**
**8b**	3.82 ± 0.37	3.49 ± 0.32	4.34 ± 0.34	3.62 ± 0.49
**8i**	3.71 ± 0.59	3.62 ± 0.30	4.60 ± 0.51	3.74 ± 0.39
**8k**	3.91 ± 0.77	3.61 ± 0.55	5.09 ± 0.33	3.14 ± 0.20
**8l**	**2.41 ± 0.21**	**2.23 ± 0.37**	3.75 ± 0.19	2.31 ± 0.34
DOX^d^	2.24 ± 0.08	0.34 ± 0.10	2.96 ± 0.26	0.79 ± 0.07

Negative control 0.1%DMSO, no activity.

a Cytotoxicity as IC_50_ for each cell line, refers to the concentration of compound which reduced by 50% the optical density of treated cells with respect to untreated cells using the MTT assay.

b The data represented the mean of three experiments in triplicate.

c Bold values signify that the bioactivity of the compound is outstanding.

d Used as a positive control.

#### 3.2.2. Structure activity relationship

Based on the activity profile of the various compounds (Figures 2, 3, and Table 1), a structure activity relationship (SAR) was developed. For the eight alkyl chain-substituted derivatives **8a–8h**, the length of the alkyl chain appeared to have a direct impact on the anticonvulsant activity of the derivatives. By increasing the alkyl chain length, the anticancer activity decreased, and ultimately disappeared at a low concentration, and compound **8a**, which bears a methoxyl group substitution, was the most active compound. This SAR might be associated with lipid-water partition coefficients of the compounds, which affects drug hydrophobic, drug-receptor interactions, metabolism of molecules, and especially the ability to pass through the cell membrane, by which its activity is determined.

Compound **8i** was substituted with a benzyloxy group at the 6-position of the triazolobenzothiazole core, then, F and Cl electron-withdrawal groups were subsequently added to the benzyloxy group at different positions, thereby yielding compounds **8j–8o**. The activities of these derivatives with different halogen substitutions on the benzene ring were determined as follows: when comparing the derivatives with different F-substitution positions on the benzyl ring, the activity order was p-F > m-F > o-F. The activities of Cl-substituted derivatives were mostly weaker when compared to that of F-substitution, except for p-Cl, however, it was still less when compared to p-F. Compound **8o** was replaced by two chlorine atoms on the benzene ring, however, the activity decreased instead of increased. Interestingly, compound **8i**, which does not bear any substitution on the benzene ring, had good anticancer activity, which was even better when compared to that of m-F substitution against HepG 2.

#### 3.2.3. Annexin V/PI flow cytometry assays

**Figure 4.**
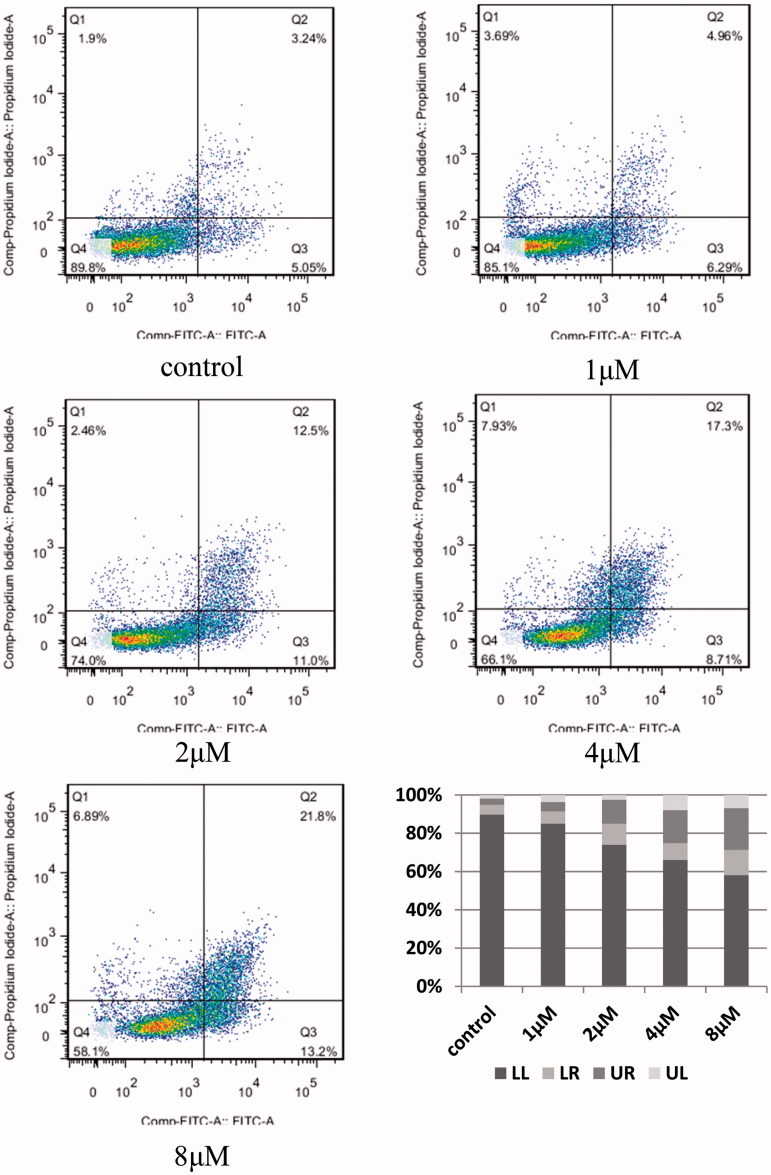
Flow cytometry analyses of apoptosis induction in triple-negative breast cancer cell MDA-MB-231 after treated by different concentrations of compound **8l** (1, 2, 4 and 8 μM) and no treatment (control) as a reference control for 48 h.

Based on the data presented above, compound **8l** showed the highest antiproliferative activity when compared to all other synthesised compounds, and was equal to the activity of Doxorubicin using MDA-MB-231 cells. To explore whether compound **8l** had the ability to induce apoptosis in MDA-MB-231 cells, we used Annexin V-FITC and PI to stain cells and evaluated the staining by flow cytometry analysis. The results are depicted in Figure 4. MDA-MB-231 cells treated with 1 µM of compound **8l** for 48 h showed an increase in the percentage of Annexin-V-positive cells, from 8.06% in control cells to 11.29% in treated cells (6.29% of cells in early apoptotic cells and 4.96% in late apoptotic cells). After increasing the concentration of the drug to 2 µM, 4 µM, and 8 µM, the percentages of Annexin-V-positive cells increased to 23.5%, 26.1%, and 35.0%, respectively. Thus, our results suggested that compound **8l** induced apoptosis in MDA-MB-231 cells in a concentration-dependent manner.

#### 3.2.4. Western blot analysis

**Figure 5.**
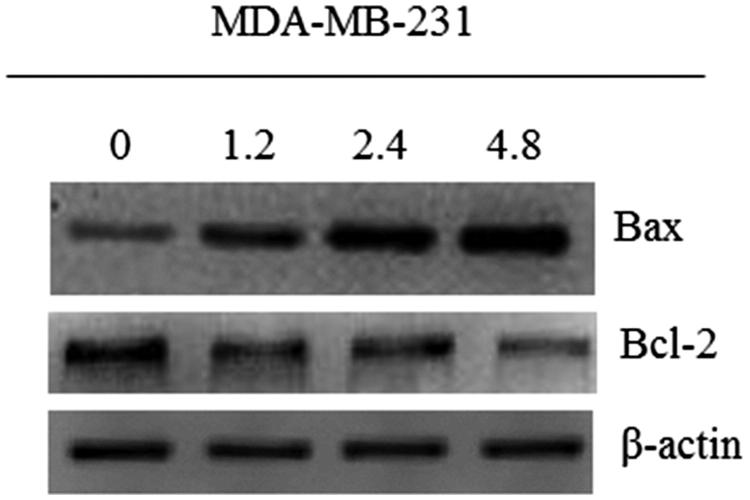
Determine the translation of proteins by western blot. MDA-MB-231 cells were treated with compound **8l** of 1.2 μM (half IC_50_), 2.4 μM (IC_50_), 4.8 μM (double IC_50_) and no treatment for 48 h, respectively. β-actin was used for equal loading.

To verify whether the compound **8l**-induced cell death observed in MDA-MB-231 cells was a result of apoptosis, Western blot analyses were conducted to determine the level of apoptosis proteins. Apoptosis was induced in MDA-MB-231 breast cancer cells by compound **8l** as confirmed by the downregulation of Bcl-2, and up-regulation of Bax protein levels (Figure 5), thereby indicating that compound **8l**-induced cell death was related to apoptosis.

## Conclusion

In this study, a novel series of 2-((1-(6-alkoxybenzo[*d*]thiazol-2-yl)-3-phenyl-1*H*-pyrazol-4-yl)methylene) hydrazinecarboximidamide derivatives were synthesised and evaluated for their antitumor activity. The results showed that all compounds showed different degrees of actives in the antiproliferative assays against several cancer cell lines. Compound **8l** exhibited potent activity against triple-negative MDA-MB-231 breast cancer cells, non-triple-negative MCF**-**7 breast cancer cells, and two types of human hepatocarcinoma cell lines, HepG2 and SMMC-7721, with IC_50_ values of 2.41 µM, 2.23 µM, 3.75 µM, and 2.31 µM. Especially, **8l** displayed a remarkable activity as good as the clinically used antineoplastic drug Doxorubicin against triple-negative MDA-MB-231 breast cancer cell. The more detailed mechanistic study demonstrated that compound **8l** inhibited the proliferation of MDA-MB-231 cancer cells by inducing apoptosis by downregulating Bcl-2 and upregulating Bax protein levels. Thus, our data showed that compound **8l** may represent a potential anticancer lead compound, which will play an important role in our follow-up study.
